# Recent Advances in the DNA-Mediated Multi-Mode Analytical Methods for Biological Samples

**DOI:** 10.3390/bios13070693

**Published:** 2023-06-30

**Authors:** Lu Huang, Zhuomin Zhang

**Affiliations:** School of Chemistry, Sun Yat-sen University, Guangzhou 510006, China

**Keywords:** DNA-mediated strategy, multi-mode analytical methods, biological samples, advance

## Abstract

DNA-mediated nanotechnology has become a research hot spot in recent decades and is widely used in the field of biosensing analysis due to its distinctive properties of precise programmability, easy synthesis and high stability. Multi-mode analytical methods can provide sensitive, accurate and complementary analytical information by merging two or more detection techniques with higher analytical throughput and efficiency. Currently, the development of DNA-mediated multi-mode analytical methods by integrating DNA-mediated nanotechnology with multi-mode analytical methods has been proved to be an effective assay for greatly enhancing the selectivity, sensitivity and accuracy, as well as detection throughput, for complex biological analysis. In this paper, the recent progress in the preparation of typical DNA-mediated multi-mode probes is reviewed from the aspect of deoxyribozyme, aptamer, templated-DNA and G-quadruplex-mediated strategies. Then, the advances in DNA-mediated multi-mode analytical methods for biological samples are summarized in detail. Moreover, the corresponding current applications for biomarker analysis, bioimaging analysis and biological monitoring are introduced. Finally, a proper summary is given and future prospective trends are discussed, hopefully providing useful information to the readers in this research field.

## 1. Introduction

Nowadays, DNA is regarded as a masterpiece in the field of nanotechnology for the multifarious and programmable structures at the nanoscale [1]. Except for the basic biological carrying functions, DNA plays the prominent role as a functional chemical molecule in the synthesis, modification and assembly of nanomaterials due to the unique properties of diverse structures, easy labeling, nanoscale rigidity, specific molecule binding and metal-ion response [2,3,4]. Thus, DNA-mediated technology driven by DNA with a specific sequence or structure can be applied in different sensing analyses to achieve selective response to the targets via various nucleic acid chain reactions [5,6,7,8] due to its distinctive properties of precise programmability, easy synthesis and high stability.

In the early 1990s, Mirkin and his co-workers [9,10] first attempted to assemble nanoparticles to form the well-defined predetermined geometric structures by using DNA oligomers. In 1992, Walker’s group [11] developed the strand displacement amplification (SDA) technique and achieved 10-fold amplification efficiency for *Mycobacterium tuberculosis* in 2 h by the strand displacement activity of the exo-Klenow fragment. In 2004, Dickson’s group [12] first synthesized silver nanoclusters (AgNCs) based on the DNA templates and then initiated the extensive exploration and application of DNA metal nanoclusters. After that, an increasing number of sensing methods coupled with the DNA-mediated technologies were well developed by assembling DNA onto the specific nanomaterials with the help of some DNA software for designing and predicting the DNA structures, especially in recent years [13,14,15]. For example, Zhang et al. [16] utilized the optimized DNA AgNCs to develop a fluorescence (FL) sensing strategy by transforming the information encoded into different DNA sequences. The proposed probes provided a simple and label-free platform for multiplexed DNA detection with a low detection limit of 25 nmol/L. Xu et al. [17] developed an integrated FL probe based on the SDA strategy to produce signal with numerous G-quadruplexes for the sensitive detection of DNA methyltransferase activity. Although the development of sensing methods has been accelerated due to the selective recognition function of DNA director, the practical applicability and high-throughput detection of single-mode analysis still hinders the analytical efficiency and accuracy for multiple trace targets.

In recent years, the multi-mode analytical methods have attracted massive attention across many different scientific fields, such as multiplex quantification analysis, optical encoding and bioimaging, etc. [18,19,20,21,22]. Compared to single-mode analytical methods [23,24,25], multi-mode analytical methods take the advantages of their multiplex ability to improve the analytical throughput and efficiency; provide sensitive, accurate and complementary analytical information; and expand the applicability by integrating or merging two or more analytical technologies [26,27]. However, when facing real biological samples with a complex matrix, the potential serious matrix interference and non-specific adsorption will greatly lower the selectivity and accuracy of multi-mode analytical methods in most cases. Thus, many reported multi-mode analytical methods have been considered more suitable for qualitative or semi-quantitative analysis and are rarely used for quantitative analysis [28]. It still remains a great challenge to develop sensitive and accurate multi-mode analytical methods with a good recognition performance and strong anti-interference ability for the rapid, sensitive and accurate analysis of trace biotargets in complex samples. Introducing DNA-mediated technology to prepare the excellent multi-mode probes and further construct the corresponding multi-mode analytical methods will improve analytical efficiency and accuracy due to the good recognition performance, strong anti-interference ability and sensitive response.

**Figure 1:**
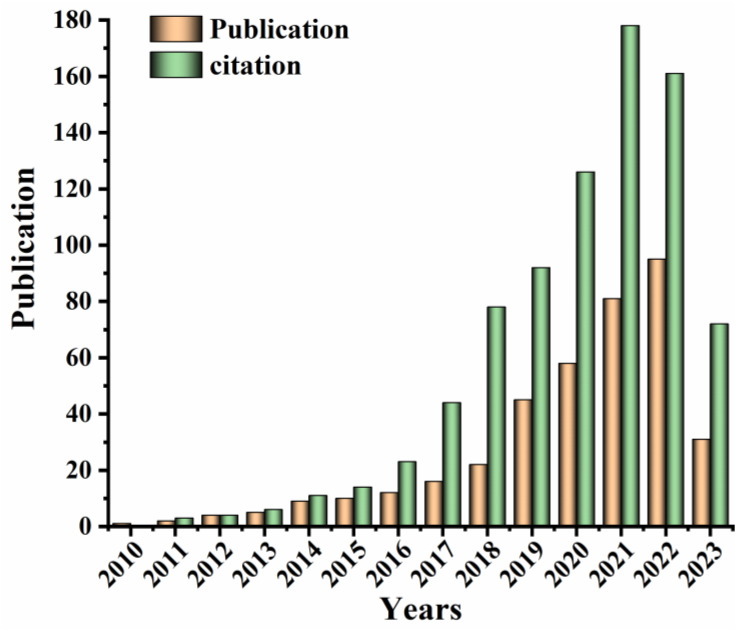
The trends of publication and citation according to the searching keywords “DNA-mediated” and “dual-/multi-mode analysis” and “biological analysis”. The data collected are from 2010 to April 2023, according to the Web of Science.

Using the DNA-mediated multi-mode analysis has become a trend since it can effectively integrate various recognition and response units through reliable multi-mode composite strategies. Integrating DNA structure to construct accurate, selective, sensitive and efficient multi-mode nanoprobes will improve the anti-interference capability, specificity, sensitivity and accuracy of multi-mode analytical methods, as well as the detection throughput [29,30,31]. At present, common DNA-mediated multi-mode analytical methods include surface-enhanced Raman scattering (SERS)/FL [32], SERS/colorimetric [33], FL/colorimetric [34], FL/photothermal (PT) [35] and SERS/electrochemistry (EC) [36], SERS/FL/colorimetric [37], SERS/resonant Rayleigh scattering (RRS)/FL [38] methods, etc. It has become a popular research interest to utilize the characteristics of DNA to design sensitive, reliable and efficient multi-mode DNA-mediated analytical methods in recent years. To date, many sensitive and efficient DNA-mediated multi-mode analytical methods have been developed and widely applied for biological analysis. Figure 1 shows the gradually increasing trends of related publications and citations regarding the DNA-mediated multi-mode analytical methods for biological samples in the last decade. Therefore, it is essential to systematically review DNA-mediated multi-mode analytical methods with different response probes, multi-mode functions and applications for complex biological samples.

This review summarizes an overview of the recent progress in DNA-mediated multi-mode analytical methods for biological samples. Firstly, the advances made in the preparation of various DNA-mediated multi-mode probes in recent years are reviewed in detail. Then, the progress made in the diverse DNA-mediated multi-mode analytical methods is systematically summarized. After that, the corresponding applications for biological samples are introduced. Finally, the challenges and future perspectives in this field are discussed and proposed.

## 2. Preparation of DNA-Mediated Multi-Mode Probes

DNA as a double-helix molecule formed by winding two complementary nucleotide chains has the characteristics of structural programmability and high stability [39]. DNA-mediated multi-mode probes as the core of DNA-mediated multi-mode analytical methods are usually prepared by integrating various sensing modules with DNA-mediated strategies into one sensing platform. Since the arbitrary combination and arrangement of DNA sequence has a great impact on its secondary structures, it provides more possibilities for the design of DNA-mediated multi-mode probes with diverse structures and properties, such as the core–shell, satellite and spindle probes [40,41,42], and further, the construction of related analytical methods. Nowadays, according to the response principles, DNA-mediated multi-mode probes can be mainly divided into deoxyribozymes (DNAzymes)-mediated, aptamer-mediated, template-DNA-mediated and G-quadruplex-mediated multi-mode probes.

### 2.1. DNAzymes-Mediated Multi-Mode Probes

The DNAzyme, as a kind of important single-strand DNA molecule, has attracted much attention due to its high catalytic activity, recognition capability and suitable stability, easy preparation and functionalization [43]. Since the DNAzyme has an excellent recognition capability for metal ions, it generally shows a specific response to many ions, such as Pb^2+^ [44], Cd^2+^ [45], Mg^2+^ [46], Ag^+^ [47], etc., which can usually be designed and prepared through the systematic evolution of ligands by exponential enrichment (SELEX) screening technology via a one-step or multi-step assembly method.

**Figure 2:**
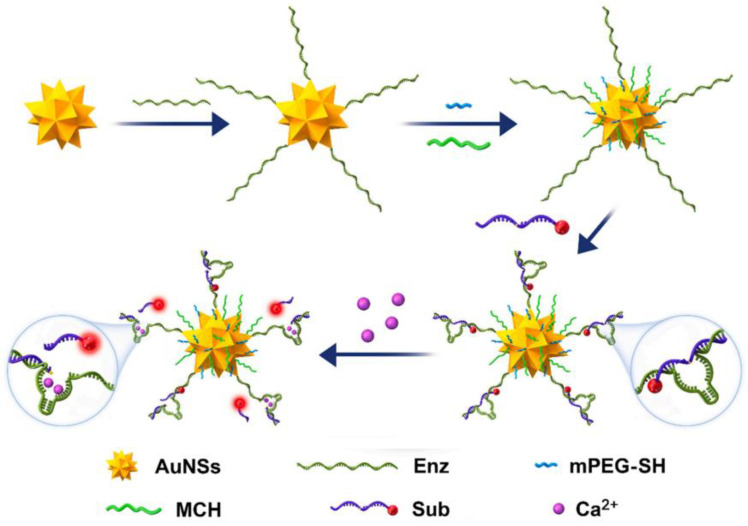
The preparation of surface-enhanced Raman scattering/ fluorescence (SERS/FL) nanoprobe based on DNAzyme-AuNSs. Reproduced with permission from Ref. [49]. Copyright 2021, Elsevier. AuNSs, Enz, mPEG-SH, MCH and Sub mean gold nanostars, enzyme chain, mercapto-1-hexanol, methoxy polyethylene glycol-thiol, substrate chain, respectively.

Introducing DNAzymes into the construction of multi-mode probes usually achieves both good specificity and fast response performance. Moreover, a good analytical performance cannot be achieved without the support of nanomaterials. Multi-step assembly methods are usually required to prepare ideal multi-mode analytical methods according to the properties of different materials [48]. Li et al. [49] prepared highly selective Ca^2+^-dependent-DNAzyme immobilized gold nanostars (AuNSs) through the Au-S bonds, as shown in Figure 2. Based on the Förster resonance energy transfer (FRET) and localized surface plasmon resonance (LSPR) effect, the feasibility of switchable FL and Raman signals was decided by the distance changes between AuNSs and reporter Cy5, which was related to the hybridization state of substrate chain (Sub) and enzyme chain (Enz) in DNAzyme with the good reactivity to Ca^2+^ in the environment and cell samples. The proposed nanoprobe with good SERS activity has been used for the fast and sensitive quantification of Ca^2+^ with limits of detection (LODs) of 0.056 μmol/L in the FL mode and 0.021 μmol/L in the SERS mode. Furthermore, a DNAzymes-mediated multi-mode strategy could improve the probe stability and reduce the matrix interference of biological samples by integrating with the enzyme-based isothermal signal amplification methods [50]. Xue et al. [51] developed a sensitive and efficient EC/FL biosensing method via enzyme-free dynamic DNA amplification to form Mg^2+^-specific DNAzyme junctions for nucleic acids’ detection, achieving LODs for EC and FL of 0.022 fmol/L and 3.51 fmol/L, respectively. This work depended on the cascaded signal amplification units that consisted of the upstream-catalytic-hairpin-assembly-mediated formation of Mg^2+^-dependent three-way DNAzyme junctions and the downstream-hybridization-chain-reaction-mediated formation of a Mg^2+^-dependent DNAzyme. Though this method was able to provide the free switching of dual-mode signals, the sensing system, which had a complicated amplification process, was prone to interference from the matrix environment of the serum sample.

The DNAzymes-mediated multi-mode probe has a fast response and high affinity for its target, along with the high catalytic activity [43,49]. However, the current analytical applications of DNAzymes-mediated multi-mode probes mainly focus on specific small molecules or ions. In the future, it will be necessary to improve the stability of some DNAzymes, prepare more types of DNAzymes-mediated multi-mode probes and widen the analytical ranges for different target molecules.

### 2.2. Aptamer-Mediated Multi-Mode Probes

A nucleic acid aptamer is a kind of oligonucleotide short chain with high affinity that can specifically bind with nucleic acid [52], protein [53], small biomolecules [54], pathogenic bacteria [55] and other substances [56]. Aptamer-mediated multi-mode probes could be achieved by connecting different functional units with good activity and response performance via the complementary pairing principle due to the excellent affinity for target molecules. At present, the preparation of aptamer-mediated multi-mode probes mainly includes the synthesis of a substrate probe; the modification of a functional aptamer; and the assembly between different response modules with various structures, such as satellite [57], spindle [58] and reticular [59] structures.

**Figure 3:**
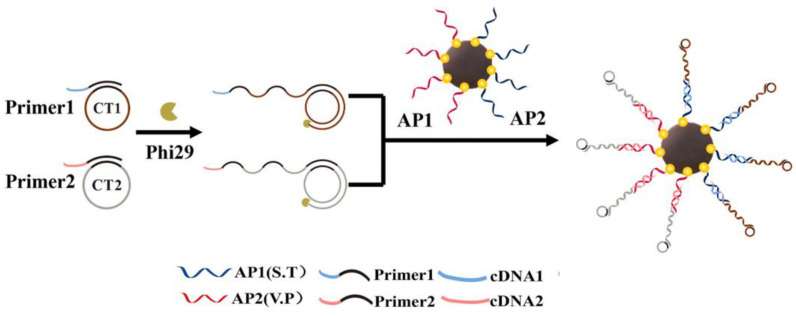
Preparation of the dual-mode aptasensor based on Fe_3_O_4_@Au-aptamer 1/aptamer 2 (Ap1/Ap2). Reproduced with permission from Ref. [61]. Copyright 2021, Elsevier.

Yu et al. [60] separately synthesized cDNA-modified gold nanoclusters (cDNA-AuNCs) and a Ag-nanoparticles-modified metal–polydopamine framework (AgNPs/MPDA) via a solvent method for the preparation of a satellite-structured SERS/FL aptasensing method. The work introduced polydopamine modified on zeolitic imidazolate framework-8 (ZIF-8) that could prevent the aggregation of Ag NPs to enhance FL quenching efficiency and generate richer SERS “hot spots”, thus greatly improving the detection sensitivity. The dual-mode method was applied for the detection of deoxynivalenol (DON) and achieved LODs of 0.08 ng/mL by SERS and 0.06 ng/mL by FL. Yu et al. [61] synthesized the magnetic-aptamer-encoded probes via a rolling-circle amplification method, which was combined with naked-eye observation and a microfluidic chip (MC) assay, as shown in Figure 3. The method showed a fast semi-quantitative analysis by the naked eye and precise quantification by MC for the simultaneous dual-mode analysis of two bacteria and achieved an LOD of 30 CFU/mL for *Vibrio parahaemolyticus* and 32 CFU/mL for *Salmonella typhimurium*, respectively. The work achieved a fast semi-quantitative analysis of multiple biotargets by the naked eye and precise quantification by MC, using the magnetic enrichment. Ali et al. [62] synthesized the functional copper-based metal–organic framework (Cu-MOF) with fluorescent enzyme mimetics via a coordination interaction and then modified the aptamer on the surface as a molecular-recognition element to prepare a colorimetry/FL aptasensing probe. This method possessed good enzymatic activity and specific binding property for thrombin detection in serum samples, which effectively avoided the assembly difficulty of various signal indicators and greatly improved the sensitivity based on the quenching mechanism. Li et al. [63] synthesized a terbium-metal–organic-framework-loaded gold nanoparticles catalyst (TbMOF@Au NPs) via a microwave method and then developed the SERS/RRS/surface plasmon resonance (SPR) probes for malathion detection. In this system, the RRS and SPR signal were obtained at the peak of 370 nm and 550 nm, respectively, based on the high SERS activity of TbMOF@Au which was achieved by the reduction of HAuCl_4_ with cysteine (Cys) to generate AuNPs on the surface. The SERS signal was generated on the enhancement effect of TbMOF@Au on the reporter molecule, which could be partially reduced due to the surface-active sites of TbMOF@Au by the block effect of the addition of AptDNA. This method could achieve both qualitative and quantitative analyses, ensuring both analytical efficiency and accuracy. The LOD for the SERS mode could be down to 0.21 ng/mL.

It can be seen that aptamer-mediated multi-mode probes have advantages of various types and easy preparation, along with a stable structure. Their response signals based on different response principles can be mutually cross-verified to improve the analytical accuracy. However, even if the aptamer-mediated multi-mode probes possess good specificity and a good recognition ability toward the targets, the severe biomatrix will interfere with the analysis in some cases. In the future, it will be a hot spot for developing diverse synthesis methods for aptamer-mediated multi-mode probes to achieve large-scale production and high-throughput detection for complex biological samples.

### 2.3. Templated DNA-Mediated Multi-Mode Probes

DNA is regarded as a molecular bio-template for the fabrication of various nanopatterns, owing to its special features, including the recognition ability, design flexibility, easy handling and self-assembly properties [64]. Moreover, DNA with different secondary structures has various degrees of rigidity. For example, the single-strand (ss) DNA has weak rigidity and is easily adsorbed on the surface of nanoparticles, while the dsDNA and hairpin DNA could maintain an upright state on the surface of nanoparticles to achieve the relatively rigid structure [65]. Introducing DNA molecular templates for the preparation of multi-mode probes can effectively regulate the generation of functional nanoprobes and further improve their recognition capability for the target. The preparation of templated DNA-mediated multi-mode probes is usually achieved based on the one-pot method and chemical cross-linking method.

**Figure 4:**
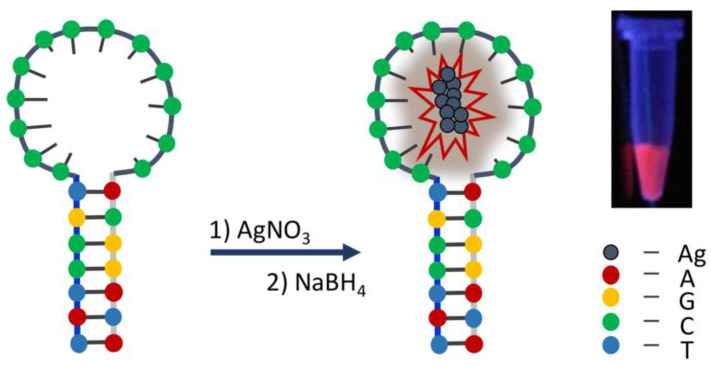
Synthesis of DNA-templated fluorescent silver nanoclusters. Reproduced with permission from Ref. [68]. Copyright 2021, Multidisciplinary Digital Publishing Institute.

Krasnoslobodtsev’s group synthesized various templated DNA-mediated metal nanoclusters with FL properties and antibacterial activity [66,67,68,69], such as the functionalized AgNPs prepared by the hairpin-DNA-templated molecule [68], which is shown in Figure 4. It could be indicated that subtle changes in the number and type of DNA bases would directly affect the FL intensity of nanoclusters, which could be used to precisely regulate the sensing performance. The proposed multi-mode method showed the advantage of high selectivity and accuracy with LODs of 23 µM in the FL mode and 29 µM in the EC mode. Li et al. [70] synthesized DNA-templated copper nanoclusters (CuNCs) via a chemical cross-linking method and then prepared the FL-colorimetric functional probes with good stability and biocompatibility. The proposed multi-mode analytical method was based on the principle of GOx catalyzing glucose to produce H_2_O_2_, which showed excellent affinity and fast response for trace H_2_O_2_ and glucose in human serum, with the related LODs being 0.266 μmol/L and 2.92 μmol/L, respectively. Except for the small biological molecules, templated DNA-mediated multi-mode probes can also be used to distinguish nuclease activity. Qing et al. [71] synthesized stable DNA-conjugated AuNCs FL/EC probes under mild conditions via a coordination reaction, using suitable DNA molecules as the template. The proposed multi-mode analytical method using these probes with good stability and sensing performance was used to determine the activity of micrococcal nuclease and identify *Staphylococcus aureus*, with LODs of 1.0 mU/mL for FL and 1.2 mU/mL for EC, respectively.

Generally, templated DNA-mediated multi-mode probes prepared based on diverse metal clusters possess special performances, such as superior affinity, biocompatibility, water solubility and high-FL quantum yields, contributing to the construction of simple and highly selective multi-mode analytical methods. Nonetheless, these probes sometimes suffer from a single type of synthesis, structural instability and susceptibility to interference from ionic media. In the future, it will be necessary to further study the binding potential of DNA templates with other functional nanomaterials to develop more types of stable multi-mode probes that adaptable for complex biological samples.

### 2.4. G-Quadruplex-Mediated Multi-Mode Probes

G-quadruplex is a special high-level structural DNA sequence formed by the tandem folding of large numbers of oligonucleotides or deoxyribonucleotide sequences that are rich in repeated guanine G [72]. Due to the different structural information and folding paths of G-quadruplex, it can not only guide the synthesis of nanoprobes but also carry out self-assembly recognition response to the target by virtue of its driving effect, which is usually used to construct multi-mode probes with excellent specificity and sensitivity [73]. Since G-quadruplex has abundant guanine G bases with the special coordination effect for K^+^ ions, it can selectively recognize K^+^ and spontaneously form a structurally stable G-quadruplex DNAzyme with Hemin (HM) via the DNA self-assembly method [74].

**Figure 5:**
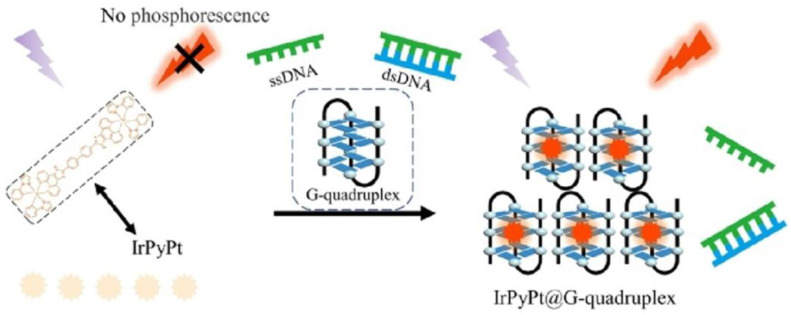
Interaction illustration between Ir^3+^-based N-heterocyclic complex (IrPyPt) and the G-quadruplex and change in phosphorescence emission. Reproduced with permission from Ref. [77]. Copyright 2023, American Chemical Society.

Chen et al. [75] utilized AgNCs and G-quadruplex/hemin DNAzyme to synthesize DNA/AgNCs probes through a self-assembly method. The developed label-free FL-colorimetric dual-mode sensing method with the available recycling and selective recognition was applied for the determination of trace human papilloma virus (HPV) with an LOD of 5.4 × 10^−16^ mol/L. In this system, the interfacial electrons from DNA/AgNCs moved to the hemin in G-quadruplex under the excited state, resulting in a sensitive FL quenching performance and significant enhancement of the absorbance intensity to the HPV target in human blood and urine samples. Jiang’s group [76] prepared the simple and rapid SERS-FL dual-mode probes with strong catalytic activity via a DNA self-assembly method for the detection of trace K^+^ and HM in urine samples with LODs of K^+^ and HM as 0.004 nmol/L and 1.6 nmol/L by SERS and as 0.88 nmol/L and 9.4 nmol/L by FL, respectively. Later, Li et al. [77] constructed the G-quadruplex-mediated phosphorescence/electrochemiluminescence (ECL) probes based on the Ir^3+^-based N-heterocyclic complex via the solvent method and DNA self-assembly, as shown in Figure 5. The developed label-free dual-mode sensing method with the available recycling and selective recognition was applied for the determination of trace miRNA-21 in cancer cell lysates with LODs of 1.40 pmol/L by phosphorescence and 0.18 pmol/L by ECL.

The preparation methods of G-quadruplex-mediated multi-mode probes are mainly based on the self-assembly integration of the G-quadruplex structure and different nanoparticles via the π-π effect, which have been widely used in EC [78] and colorimetric [79] assay systems. Currently, G-quadruplex-mediated multi-mode probes with good sensitivity and accuracy are used for biomarker analysis in biosamples with a simple matrix. In the future, it will be necessary to further improve the probe stability and enlarge the practical application range by developing functionalized multi-mode probes with selective enrichment and separation performance.

## 3. Development of DNA-Mediated Multi-Mode Analytical Methods

The multi-mode analysis has attracted widespread attention from analysts for the development of various methods in recent decades [80,81,82]. Compared with the single-mode analysis, the multi-mode analytical methods have higher detection throughput and a better identification performance [83]. In recent years, various DNA-mediated strategies have been introduced to prepare multi-mode probes and further develop DNA-mediated multi-mode analytical methods, which greatly enhance the recognition specificity, analytical precision and accuracy. Currently, common DNA-mediated multi-mode analytical methods mainly include SERS-based, FL-based, EC-based and colorimetric-based multi-mode methods.

### 3.1. SERS-Based Multi-Mode Methods

SERS is an inelastic molecular vibration scattering spectroscopy technology with unique fingerprinting characteristics that reflect the spectral information of molecular structure and composition, and it was one of the most important technologies for developing the multi-mode analytical methods [84]. SERS reporters are usually the molecules with large Raman cross-sections and can be easily bound onto metal nanoparticles via thiol bonds. For example, dye molecules as SERS reporters can be flexibly bound on the metallic nanostructure surface by controlling DNA sequences for efficient switchable multi-mode signals [85]. Since the SERS mode is typically more sensitive as compared with alternate approaches in multi-mode methods, this part focuses on the development of SERS-based multi-mode analytical methods.

**Figure 6:**
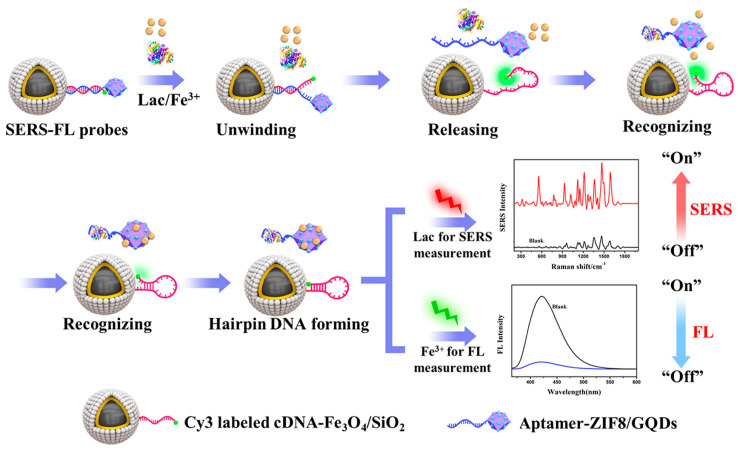
The SERS/FL dual-mode analysis of lactoferrin (Lac) and Fe^3+^. Reproduced with permission from Ref. [88]. Copyright 2021, American Chemical Society.

Lin et al. [86] fabricated a SERS/up-conversion luminescence (UCL) sensing platform based on up-conversion nanoparticles (UCNPs) and gold nanourchins (GNUs) with superior charge transferability. When the adapter hybridized with its complementary DNA, abundant electrons were transferred from UCNPs to labeled organic dye molecules through the FRET effect, resulting in the reduction of FL signals and enhancement of Raman signals due to the proximity of GNU particles. The proposed dual-mode method was applied for the sensitive detection of ochratoxin A (OTA) in a beer sample with an LOD of 8.6 pg/mL in SERS mode and 3.2 pg/mL in UCL mode. Zhang et al. [87] developed a SERS/FL nanosensing method with one binding site of aptamer based on gold nanotriangles (AuNTs) for the trace analysis of cytochrome c (Cyt c) in living cells. The proposed SERS/FL dual-mode method showed good selectivity and sensitivity for quantification and imaging analysis of Cyt c, achieving an LOD of 0.02 μmol/L in the SERS mode. Recently, our group [88] developed a hairpin DNA-mediated SERS/FL dual-mode method based on the AgNPs-loaded magnetic nanospheres-ZIF8 and graphitic quantum dot composite (Fe_3_O_4_/Ag-ZIF8/GQD) NPs for the simultaneous determination of lactoferrin (Lac) and Fe^3+^ in complex samples, as shown in Figure 6. The proposed method could quickly recognize the target via the aptamer and achieved LODs of 0.14 μg/L for Lac by SERS and 3.8 nmol/L Fe^3+^ by FL. This work provided an effective tool for improvement of the detection throughout through the simultaneous detection of two targets in different modes via a flexible DNA-mediated design. Li et al. [89] constructed an aptamer-mediated colorimetric/SERS dual-mode analytical method based on the peroxidase-like activity and SERS effect of AuNPs/Cu TCP (Fe). The method was employed for the detection of trace levamisole (LEV) in milk samples. The LODs for LEV were 5 nmol/L in the colorimetric mode and 1.12 nmol/L in the SERS mode. Liu et al. [90] proposed a sensitive SERS/colorimetric aptasensing method using the trans-cleavage activity of clustered regularly interspaced short palindromic repeats (CRISPR)/Cas12a for the on-site detection of nucleic acid. The dual-mode method showed the good response for target DNA with the two signal-reporters-loaded liposomes, which could achieve the satisfied LODs of 100 amol/L in the SERS mode and 10 pmol/L in the colorimetric mode, respectively.

SERS-based multi-mode analytical methods are gradually becoming popular for multiple and ultra-sensitive qualification and quantification of biological targets due to the fingerprint spectral information provided and flexible combination with other analytical technologies. However, the SERS-based multi-mode probes usually suffer from a complicated fabrication process and unstable binding structures in a complex matrix. Thus, it is necessary to further develop efficient and feasible strategies and enhance the reliability and anti-interference performance of the SERS-based multi-mode analytical methods in the future.

### 3.2. FL-Based Multi-Mode Methods

Due to the excellent sensitivity, simplicity and efficiency of FL spectroscopy, FL analysis has been proved to be a promising method for qualitative or quantitative analysis for various targets [91]. Furthermore, it exhibits high throughput, easy operation and enough spatial resolution for bioimaging by collecting intensity information of two-dimensional or three-dimensional FL signals and revealing the morphological characteristics of typical biological samples. DNA-mediated FL-based multi-mode analytical methods can achieve fast and sensitive determination of biotargets by use of various FL materials with excellent optical performance, as well as the in situ distribution characteristics of species and contents of biotargets at the intracellular organelle level, with better resolution via corresponding FL imaging technologies within a few seconds in most cases, thus greatly expanding the application range of the FL-based multi-mode analysis. Thus, this part of the paper focuses on introducing the development of FL-based multi-mode analytical methods.

Recently, our group [92] constructed a SERS/FL sensing platform using AuNPs loaded on the surface of magnetic Fe_3_O_4_ nanoparticles with the good selective recognition ability by the sandwich structure of two binding sites. The proposed dual-mode method could achieve the quantification of vascular endothelial growth factor (VEGF) by SERS with an LOD of 2.3 pg/mL and obtain the distribution information in living cells by FL imaging. Integrating FL and other imaging technologies to prepare multi-mode probes has attracted great interest in various fields, such as biomedicine diagnostics, biochemical detection and bioimaging analysis, and even in clinical therapeutics. Wang et al. [93] designed a kind of FL/magnetic resonance (MR) dual-imaging probes based on SiO_2_@C-coated magnetic nanoparticles with high biocompatibility. By integrating overexpressed MUC1 protein with the aptamer, the dual-imaging method had good sensitivity for capturing and quantifying the circulating tumor cell (CTC). The result indicated that the proposed method could detect CTC in blood with an LOD of 5 cells/0.15 mL in FL mode, showing great potential for the early stage tumor detection.

Furthermore, the selectivity and stability of FL-based DNA-mediated multi-mode analytical methods have gained the attention of some researchers. Shin et al. [94] utilized the stabilized hairpin-structure DNAzymes to prepare graphene oxide (GO)-based FL/colorimetric probes through a one-step method. The developed dual-mode method was employed for miRNA-21 detection with good binding affinity and achieved LODs of 0.79 nmol/L in the FL mode and 2.60 nmol/L in the colorimetric mode. Furthermore, multi-mode imaging technology using corresponding probes is promising for cancer phototherapy treatment. Singh et al. [95] developed an FL/two-photon (TP)/Raman triple-mode imaging method based on the functionalized carbon dots (CDs) with FL mesoporous bioglass nanoparticles (fBGns) modified on the surface. Due to the high surface area of CD-based fBGns, this probe achieved a high drug-loading capacity for delivery service and PT conversion treatment for tumor cells. The results of the in vivo imaging analysis in different optical modes indicated that the pH-dependent triple-mode nanoprobe had a quick and effective performance regarding the drug delivery of doxorubicin (DOX).

**Figure 7:**
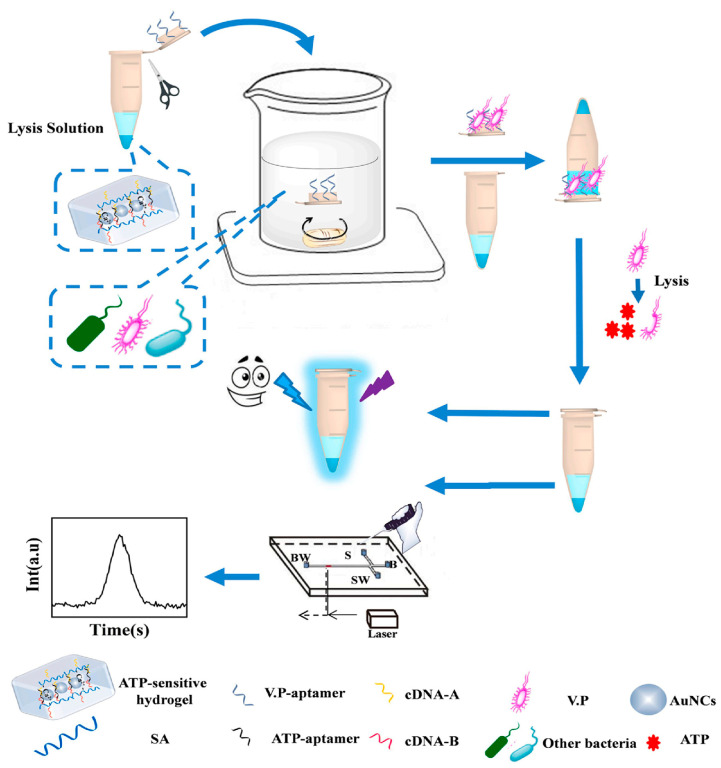
The fluorescence/microfluidic chip (FL/MC)dual-mode aptasensing method based on ATP-sensitive hydrogel for detection of *Vibrio parahaemolyticus* (V.P.) Reproduced with permission from Ref. [99]. Copyright 2020, Elsevier.

On the other hand, some advanced platforms or devices such as microfluidic chip (MC) [96], smartphone [97] and three-dimensional (3D) printing [98] were introduced to DNA-mediated FL-based multi-mode methods to enhance the convenience of the analytical process, reduce the analytical cost and improve analytical efficiency. Yu et al. [99] prepared an adenosine triphosphate (ATP)-sensitive smart hydrogel dual-mode aptasensing method based on gold nanoclusters (AuNCs) for quantifying *Vibrio parahaemolyticus* analysis, as shown in Figure 7. In this work, the target pathogen could be enriched by aptamer-modified lid and was easily cracked with the added lysozyme to produce the contained ATP. The cross-linked aptamer was combined with ATP in the hydrogel, followed by the breakdown of the SA-A-aptamer-B-SA duplex and the destruction of the hydrogel structure, resulting in the release of AuNCs and ATP-aptamer complex. The FL/MC method introduced the enrichment and separation units and has been successfully applied for the analysis of target in water and fish samples with LODs of 100 CFU/mL in FL mode and 10 CFU/mL in MC mode within a short time. Li et al. [100] synthesized porous CuNC-loaded ZrO_2_ nanoparticles (ZrO_2_@CuNCs) as triple-mode probes to significantly enhance the intensity and stability of this system. Based on the inner filter and dynamic quenching effect, the proposed FL/colorimetry/smartphone method was applied to determine metoprolol tartrate (MPT) in serum and urine samples with satisfactory results. Wang et al. [101] prepared the bifunctional iron and cobalt co-doped carbon quantum dot (Fe,Co-CQD) as a ratiometric FL/colorimetric sensing platform with good optical properties and peroxidase-mimetic catalytic activity. This dual-mode sensing platform was constructed for the analysis of glucose at physiological pH with LODs of 0.167 μmol/L by FL and 1.126 μmol/L by colorimetry.

DNA-mediated FL-based multi-mode analytical methods with good quantitative and imaging capabilities have shown a great analytical performance in complex bioanalysis. However, because of their weak stability and spontaneous FL background interference, developing more FL-based multi-mode analytical methods and platforms with improved affinities and biocompatibility for sensing applications in biological analysis is still a great challenge.

### 3.3. Electrochemistry-Based Multi-Mode Methods

Recently, the DNA-mediated EC-based multi-mode method has gradually become a popular multi-mode analysis option due to the excellent selectivity and stability of the EC assay. It is particularly suitable for the analysis of small-volume biochemical samples by judging the accuracy and reliability of the results from two or more detection channels, thus avoiding false-positive or -negative analytical results. Moreover, EC, as a rapid analytical method, has high sensitivity and a fast detection speed with simple convenient instruments and can be easily integrated with other sensing methods to develop EC-based multi-mode methods. Due to the design flexibility and signal-amplification effect, DNA-mediated strategies have been widely employed to improve the specificity of EC-based multi-mode analytical methods. On the other hand, good response performance and EC activity cannot be achieved without the support of conductive functional nanomaterials. For example, MXenes material contains a large specific surface area with -O, OH and/or -F functional groups [102], which can effectively absorb small reporter molecules such as rhodamine 6G [103], crystal violet [104] and malachite green [105] to generate an EC response. According to the different EC signals, the current EC-based multi-mode methods can be mainly classified as voltammetry, electrochemical impedance spectroscopy (EIS) and other measurements. 

**Figure 8:**
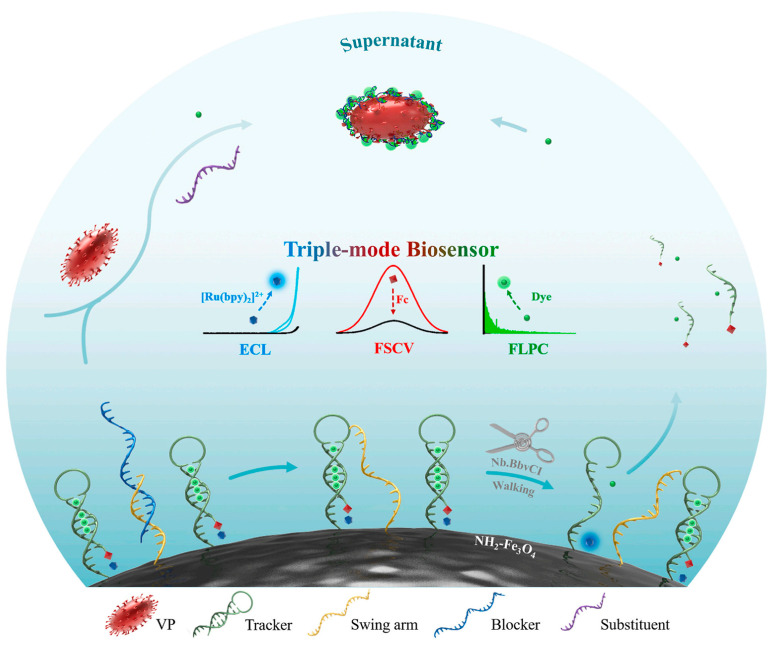
The triple-mode biosensing method with triple-signal-outputs mode for *Vibrio parahaemolyticus* detection based on the activation of DNA strands’ displacement reactions and endonuclease cleavage of DNA walker. Reproduced with permission from Ref. [108]. Copyright 2021, Elsevier.

Based on voltammetry detection, Xu et al. [106] constructed an EC/PEC dual-mode method based on the MOF and covalent organic framework (COF) hybrid material of Cu-MOF@CuPc-TA-COF with a rich amino structure, excellent EC activity and high photoactivity. The proposed dual-mode analytical method was used for the quantification of human immunodeficiency virus type 1 (HIV-1) and showed good selectivity, stability, reproducibility and a good regeneration ability with LODs by EC and PEC of 0.18 fmol/L and 0.07 fmol/L, respectively. Zhai et al. [107] proposed a fast and sensitive SERS/EC biosensing method based on molybdenum disulfide (MoS_2_) nanosheet probes and an Ag nanorods (AgNRs) array electrode for in situ dual-mode detection of gastric-cancer-related miR-106a. The dual-mode method effectively improved the response intensity and stability of EC and SERS in electrolyte solutions, with satisfactory LODs of 67.44 fmol/L and 248.01 fmol/L for SERS and EC, respectively. Later, Wei et al. [108] constructed a DNA-walker-mediated EC/fast scan cyclic voltammetry (FSCV)/fluorescent pixel-counting (FLPC) biosensing method for the sensitive and reliable detection of pathogenic bacteria, as shown in Figure 8. The conformational change of the DNA hybrid was induced by the target to activate the DNA walk on the conductive surface of Fe_3_O_4_ NPs to generate a triple signal output. The target-triggered method showed good accuracy and reliability, with LODs for *Vibrio parahaemolyticus* of 1 CFU/mL for ECL, 1 CFU/mL for FSCV and 10 CFU/mL for FLPC.

Generally, EIS is used to verify the interfacial electron-transfer performance of the modified electrodes. Based on EIS detection, Hu et al. [109] developed an EC/electro-chemiluminescent (ECL) biosensing method for Pb^2+^ analysis through the shearing action of Pb^2+^ to Pb^2+^-special DNAzyme (Pb-DNAzyme). In this work, the 3D DNA nanoprism was formed on the Au electrode surface, which provided a stable aqueous environment, the researchers adjusted the density of the DNA-mediated probe to minimize non-specific adsorption. The dual-mode method was successfully applied for the analysis of Pb^2+^ in the serum and lake-water samples with LODs of 3.6 pmol/L in the EC mode and 0.23 pmol/L in the ECL mode. Zhang et al. [110] constructed an EC/colorimetric paper sensing platform based on aptamer-modified AuNCs loaded on the surface of ZIF-8 as nanocarriers. In the presence of OTA, a free-capturing DNA structure was triggered and switched from a random conformation to a G-quadruplex; it then further formed G-quadruplex/hemin DNAzyme via self-assembly with the release of hemin. The dual-mode method provided a good binding encapsulation ability by employing the Au NCs and aptamer double-strand hybrid as linkers for OTA to enhance the sensitivity and reliability with, giving an LOD of 0.347 pg/mL for the EC mode.

At present, the DNA-mediated EC-based multi-mode method has achieved good selectivity and sensitivity, but sometimes it is influenced by matrix interference from the testing solution. It is expected that introducing adaptable, fast sample pretreatment methods to EC-based multi-mode methods will improve the anti-interference capability and further the analytical accuracy.

### 3.4. Colorimetry-Based Multi-Mode Methods

The colorimetry analysis is generally used to determine the existence of the target substance in the sample by observing the color change with the naked eyes, with the advantages of fast detection speed and no need for extra instrumental detectors. DNA-mediated colorimetry-based multi-mode analytical methods can be constructed by merging the qualitative colorimetric mode with other quantitative modes, achieving both preliminarily rapid screening and a consequent accurate quantitative analysis of biological samples [110,111,112]. Due to the excellent application convenience, colorimetry-based multi-mode methods have been widely used in biochemical analysis, greatly improving the analytical efficiency. Introducing DNA-mediated strategies to colorimetry-based multi-mode analytical methods will enhance the selective recognition for trace targets in complex biological samples. Currently, DNA-mediated colorimetry-based multi-mode analytical methods mainly include colorimetry/SERS [113], colorimetry/FL [114], colorimetry/EC [115], colorimetry/PT [116] and colorimetry/MC [117], which have been widely used for the analysis of metal ions [118], proteins [119] and pathogenic bacteria [120] in biological samples.

**Figure 9:**
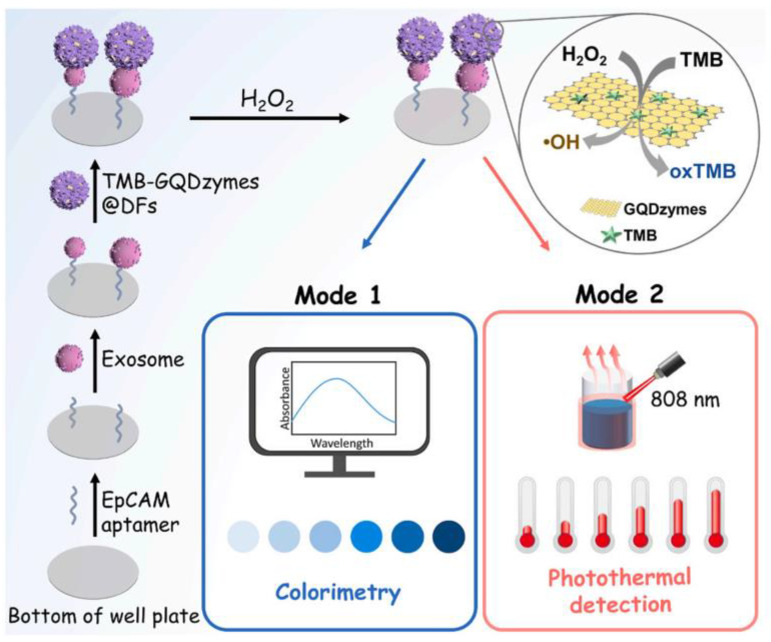
The colorimetric/photothermal (PT) dual-mode biosensing method for the detection of michigan cancer foundation-7 (MCF-7) cell-derived exosomes. Reproduced with permission from Ref. [122]. Copyright 2023, Elsevier.

Zhong et al. [121] constructed a colorimetric/SERS dual-mode analytical method based on Au@Ag NPs via the seed-growth method. The method was employed for the fast simultaneous determination of H_2_O_2_ and glucose, with LODs of 20 nmol/L in the SERS mode and 300 nmol/L in the colorimetric mode. Zhang et al. [122] encapsulated 3,3′,5,5′-tetramethylbenzidine-loaded graphene quantum dot nanozymes (TMB-GQDzymes) into DNA flowers (DFs) to prepare a colorimetric/PT exosome sensing platform via rolling circle amplification (RCA), as shown in Figure 9. In the presence of H_2_O_2_, TMB could be oxidized by GQDzymes to produce oxTMB, which exhibited an obvious absorption peak at 650 nm for a colorimetric signal and the near-infrared laser-driven photothermal effect for PT response. The proposed dual-mode method could be successfully used for the quantification analysis of human breast cancer cell (MCF-7)-derived exosomes in serum, with LODs of 1027 particles/μL in the colorimetric mode and 2170 particles/μL in the PT mode. Zhang et al. [123] prepared a fast and sensitive SERS/colorimetric aptasensor based on functional Au@Ag NPs. In the presence of the target, the dual-mode probes showed a red shift for sulfadimethoxine by the induced aggregation of nanoparticles and achieved LODs of 0.89 ng/mL in the SERS mode and 2.41 ng/mL in the colorimetric mode. Zheng et al. [124] developed a robust triple-mode sensing strategy capable of visual colorimetric/FL/SERS detection of Cu^2+^ via stimuli-responsive binaphthalene-functionalized Au nanorods. Due to the preferential affinity with Cu^2+^, the triple-mode sensing assays showed high flexibility, simplicity and sensitivity, achieving satisfactory LODs for Cu^2+^ of 3.0 nmol/L by colorimetry, 0.42 nmol/L by FL and 0.38 pmol/L by SERS.

To date, DNA-mediated multi-mode analytical methods are usually constructed based on the well-designed DNA-mediated strategies and highly active nanomaterials to achieve excellent specificity and sensitivity, as well as high analytical throughput in the field of biological analysis. The DNA-mediated multi-mode analysis can not only conduct qualitative to quantitative analysis on specific biomolecules but also further perform efficient imaging of specific molecules at the cellular or tissue level to display their biological behaviors. It is noted that some DNA-mediated multi-mode methods, such as FL/SERS imaging [125] and FL/photoacoustic/PT imaging [126], have been recently developed that can not only provide the satisfied analytical results of bio-targets but also exhibit interesting potential in clinical science. Nonetheless, when DNA-mediated multi-mode analytical methods are applied for living cell, tissue or even in vivo analysis, the continuous improvement of the anti-interference capability by introducing simple and fast sample pretreatment techniques [127] into DNA-mediated multi-mode analysis will be a potential research interest. Moreover, the enhancement of the penetration efficiency and biocompatibility of DNA-mediated multi-mode analytical methods for biological samples will be another essential point in the future.

## 4. Applications for Bioanalysis

The process of life involves dynamic changes in the conformation, distribution and interaction of various biomolecules, which are closely related to life activities and health situations. Through the reasonable design of DNA-mediated multi-mode probes with high sensitivity, excellent specificity and good biocompatibility, the corresponding multi-mode analytical methods can be effectively developed to realize the sensitive and high-throughput detection, quantification or real-time imaging of trace biomarkers and important bio-information in complex biological samples. Currently, the applications of DNA-mediated multi-mode analytical methods mainly focus on the biomarker analysis, bioimaging analysis and biological monitoring.

### 4.1. Biomarker Analysis

Various active biological small molecules and enzymes participate in diverse biochemical reactions and affect corresponding physiological processes, which are usually considered to be crucial biomarkers for the life process. The content levels of biomarkers are closely related to the physiological activity of cells or tissues and the health status of the human body. Currently, the crucial biomarkers mainly include DNA, microRNA, polypeptides, proteins, free radicals, etc.

Song et al. [128] developed a SERS/SPR dual-mode analytical method based on tetrahedral DNA and a silver nanorod-covered silver nanohole (Ag-NR-NH), which realized the highly sensitive detection of nucleic acid in serum through DNA-signal amplification. In order to obtain high sensitivity and specificity, they fixed the intelligent tetrahedral DNA probe onto the Ag-NR-NH array and combined the DNA-sandwich-sensing strategy to realize the amplification of DNA signals. The signal of SPR and SERS by this dual-mode method could be enhanced by 10 times and 4 times with LODs for the SERS and SPR modes of 0.77 fmol/L and 0.51 pmol/L, respectively, and the specificity of the SPR mode was much higher than that of the SERS mode. Li et al. [129] developed a decoupled PEC/EC bioassay for the sensitive detection of miRNA-21 with high reliability by the 3D DNA nanoring photoelectrode signal amplification strategy. The dual-mode method showed the advantages of good reproducibility and easy operation with LODs of 0.13 pmol/L in the PEC mode and 0.25 pmol/L in the EC mode, respectively. He et al. [130] constructed an LSPR/FL dual-mode probe based on fluorescent AgNCs by using mono-DNA as the etching agent. The proposed dual-mode method showed tunable LSPR and FL signals and a good analytical performance for trypsin, with an LOD of 0.20 nmol/L in the LSPR mode and an LOD of 0.12 nmol/L in the FL mode.

**Figure 10:**
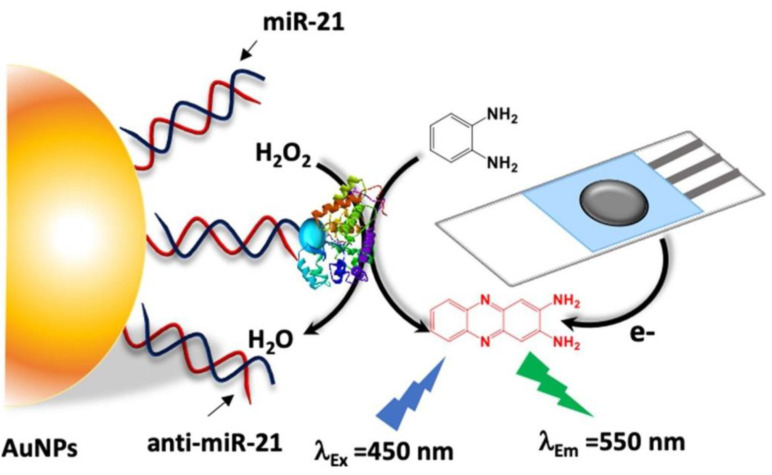
The dual-mode FL/electrochemistry (EC) bioplatform for miRNA detection. Reproduced with permission from Ref. [133]. Copyright 2021, Elsevier.

Generally, the molecular recognition efficiency of DNA is closely related to the molecular weight of the target molecules. The substances with a simple structure and small molecular weight, such as ions and small molecules, are more easily captured by DNA, while high-molecular-weight substances with complex conformation, such as polypeptide and proteins, are difficult to be captured since they can only be partially recognized by DNA [131,132]. As shown in Figure 10, Zayani et al. [133] constructed an FL/EC bioplatform via the biotinylated miRNAs and conjugation with streptavidin–horseradish peroxidase, achieving the detection of the target miRNA-21 in blood with LODs of 15 fmol/L in the FL mode and 19 fmol/L in the EC mode, respectively. In this system, the signal value reached maximum with the competitive hybridization between biotinylated miRNA (bt-miR) and target miRNA. After that, the dual-mode signal was simultaneously reduced with the addition of miRNA-21, since the number of bt-miR sequences significantly decreased, as well as the amount of streptavidin–horseradish peroxidase enzyme conjugate (Strep-HRP) immobilized on the surface of AuNPs. Dong et al. [134] prepared the thrombin (TB)-activated FL/SERS optical nanoprobe based on rhodamine-B-modified AuNPs-loaded magnetic nanoparticles. The proposed method showed good selectivity by TB-specific recognition peptides and was used to evaluate the inhibitory effect of TB in Chinese medicinal materials, which extended the detection range from 1 to 150 pmol/L, with LODs of 0.35 pmol/L for SERS and 5 pmol/L for FL, respectively. Ye et al. [135] prepared a simple FL/colorimetric/smart phone sensing platform based on silicon nanoparticles via a one-pot hydrothermal method. The proposed method could be applied for the detection of N-acetyl-β-d-glucosaminidase (NAG) and β-galactosidase (β-GAL) with LODs for NAG of 0.0086 U/L by FL and 0.96 U/L by colorimetry and LODs for β-GAL of 0.084 U/L by FL and 2.3 U/L by colorimetry.

The biomarker analysis is crucial to understand many physiological processes and further benefit the disease diagnosis and other clinical purposes. Currently, the applications of DNA-mediated multi-mode analytical methods in biomarker analysis mainly focus on the functional small biomolecules or proteins, as shown in Table 1. It still faces big challenges in practical clinical diagnosis due to the complex matrix interference and weak DNA-binding ability. Therefore, it is necessary to improve the recognition and capture ability of DNA-mediated strategies and expand other specific macromolecular metabolites as alternative biomarkers for the sake of accurate, sensitive and efficient biochemical analysis in future. 

### 4.2. Bioimaging Analysis

With the assistance of nanoprobes with high spatial resolution and a good organizational penetration ability, the bioimaging analysis is gradually becoming a key tool for understanding the physiological structure of tissues or organs and elucidating the related functions, activities and mechanisms. However, the bioimaging analysis still faces low penetration efficiency and unsatisfied resolution in some cases, thus hindering the application of bioimaging methods for complex biological analysis. By introducing DNA-mediated multi-mode methods, we expect to improve the spatial resolution and provide accurate quantification for biological imaging analysis. Currently, the applications of DNA-mediated multi-mode methods for bioimaging analysis mainly include the combination mode of quantification–bioimaging (e.g., SERS/FL [146], FL/MR [147], etc.) and multiple imaging mode (e.g., SERS/MR [148], FL/CT/MR [149], etc.).

**Figure 11:**
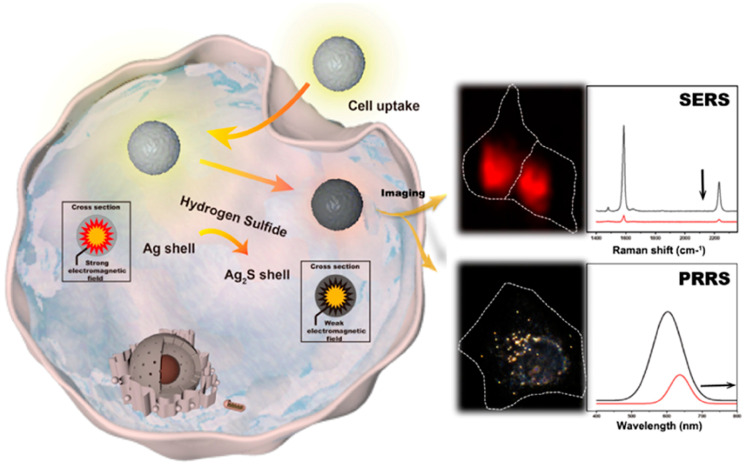
Dual-mode imaging analysis of intracellular glutataione (GSH) and enhanced chemodynamic therapy. Reproduced with permission from Ref. [150]. Copyright 2021, American Chemical Society.

The combination of quantification–bioimaging can usually achieve both quantification and distribution information of typical biomarkers in complex biological samples. Wang et al. [150] developed a SERS/plasmon resonance Rayleigh scattering (PRRS) nanoprobe based on dyes-embedded core–shell AuNSs for the real-time dual-imaging of H_2_S in living cells, as shown in Figure 11. In this work, the PRRS mode was used to study the local concentration of H_2_S in one cell, while the SERS mode was conducted to detect the average concentration of H_2_S in each cell. The multi-mode imaging method could usually provide crucial multidimensional imaging information for a biotarget analysis at different physiological statuses in the non-destructive and in vivo way. Nonetheless, the imaging resolution and precise orientation of the related lesion area at the molecular level still remain a big challenge. TP technology as a high-resolution imaging mode has attracted more and more attention for biomedical applications, which could be combined with multi-mode imaging analysis to improve the imaging sensitivity and resolution. Zhong et al. [151] prepared the TP-CDs loaded on MnO_2_ nanosheets (TP-CQDs@MnO_2_) dual-mode nanoprobe for an FL/MR imaging analysis of H^+^ in cells. The proposed method could monitor intracellular-wide pH (4.0–8.0) via the FL mode, and the MRI mode was used to clearly define its distribution in living cells. Weng et al. [152] prepared a versatile DNA-functionalized FL/PA/two-photon bioimaging platform via the self-assembly of red emission CDs and indocyanine green (ICG)-modified double hydroxides. The triple-mode probes were able to stabilize the ICG molecule through the layered structure of the nanocarrier, significantly enhancing its PT conversion efficiency and extending the FL lifetime of CDs in favor of bioimaging, which showed great potential in multi-mode system integrating diagnosis and treatment. At present, the DNA-mediated multi-mode method for bioimaging analysis has achieved preliminary application in imaging studies of small biomolecular or drug monitoring. The future development of DNA-mediated multi-mode technology in biological imaging needs to not only further improve the imaging resolution but also enhance the real-time and continuous imaging performance, achieving continuous tracking of individual biological functional molecules in vivo, recording their physiological processes in detail and fully revealing their biological functions.

### 4.3. Biological Monitoring

The physiological processes of humans mainly involve dynamic changes in the conformation, distribution and interaction of various biological molecules which are closely related to the health status and life activities of the human body [153]. Although some methods have been reported for monitoring various biomolecules, there are still some limitations that need to be tackled in terms of improving the resolution at the single-molecule scale and imaging depth with the short observation time of the dynamic biological processes as much as possible. It has been considered an effective way to improve the real-time monitoring efficiency, sensitivity and accuracy with good spatial resolution by introducing DNA mediated multi-mode analytical methods for biological monitoring [154].

Wang et al. [155] developed a DNA self-assembly nanomachine by integrating different diagnostic modes, and it was applied for aptamer-mediated SERS/FL dual-mode dynamic monitoring and phototherapy of miRNA in tumor cells. The method was capable of in situ FL imaging of tumor focus and SERS quantification of the target by the triggered dual-mode nanosystem, providing the clues for the dual-mode diagnosis and effective therapy means of the tumor. Liu et al. [156] proposed a multicolor/SERS dual-mode pH-sensing method based on polyethylene glycol diacrylate microgels. The dual-mode sensing method exhibited excellent stability, reversibility, biocompatibility and signal-enhancement ability, ensuring the successful application for pH (1.0–12.0) monitoring in the cell culture process.

**Figure 12:**
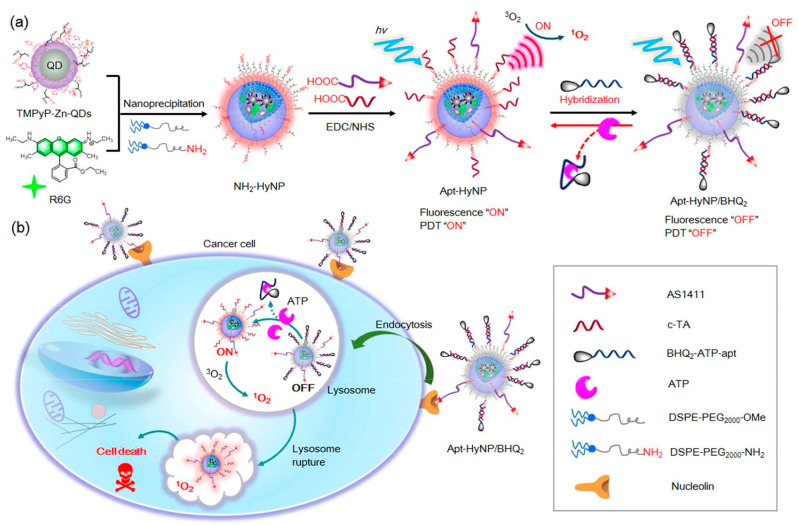
The tumor-targeting and adenosine triphosphate (ATP)-activatable aptamer-mediated quencher-labeled nanophotosensitizer (Apt-HyNP/BHQ2) for fluorescence imaging and photodynamic therapy (PDT) of tumors. (**a**) Synthesis of Apt-HyNP/BHQ2; (**b**) Action mechanism of Apt-HyNP/BHQ2 for nucleolin-mediated endocytosis and intracellular ATP-activated FL and PDT for tumor imaging and PDT. Reproduced with permission from Ref. [159]. Copyright 2017, American Chemical Society.

Furthermore, the multi-mode imaging in field of biological monitoring can also be applied to provide crucial diagnosis clues of clinical disease. Zhang et al. [157] developed SERS/FL probes based on the hairpin DNA-modified AuNPs with the reciprocal changes of dual signals for the quantification and monitoring analysis of miRNA in living cells. The proposed dual-mode imaging method could be used for the real-time assessment of intracellular miRNA dynamics as a biomarker due to the good sensitivity, which could offer the precise information for clinical diagnosis. Chen et al. [158] constructed a selective and sensitive near-infrared FL/PA probe based on the DNA self-assembly method for Cys detection. In the presence of Cys, the probe could activate near-infrared FL/PA signals with the improved emission intensity for monitoring the biomolecules in tumor cells or tissues. In addition, Shen et al. [159] constructed a dual FL imaging and specific photodynamic therapy (PDT) system for tumors based on the aptamer-mediated quencher-labeled nanophotosensitizer (HyNP/BHQ2), as shown in Figure 12. The corresponding in vitro and in vivo test demonstrated that Apt-HyNP/BHQ2 could specifically interact with endogenous ATP after selectively entering tumor cells via the nucleolin-mediated endocytosis, resulting in activation of FL and PDT efficacy. The proposed method provided highly sensitive and selective recognition ability for endogenous ATP, which had been applied in the precise monitoring of ATP levels and controlling drug release in living mice to achieve an on-demand PDT effect.

To date, the applications of DNA-mediated multi-mode analytical methods in biological analysis are mainly focused on the high-throughput and accurate acquisition of diverse bioinformation from the qualitative and quantitative analysis of specific biomarkers to their distribution imaging clues. The development of real-time and dynamic monitoring multi-mode methods is gradually becoming a hot spot in this field due to possibly providing more accurate bioinformation for the dynamic physiological process during biological analysis. It is hoped that with the development of more advanced multi-mode nanoprobes with higher specificity, anti-interference capability and biocompatibility, DNA-mediated multi-mode analytical methods will move towards broader and deeper practical clinical analysis applications.

## 5. Conclusions and Perspective

DNA-mediated technology possesses powerful direction and recognition capability for biological targets. The integration of DNA-mediated technology with multi-mode analytical technology to develop DNA-mediated multi-mode analytical methods will provide the promising potential to enhance the analytical sensitivity, selectivity and accuracy, along with with higher detection throughput for complex biological analysis. Currently, DNA-mediated multi-mode analytical methods based on various DNA-mediated nanoprobes have been widely used for obtaining the diverse and crucial bioinformation from qualitative and quantitative results of biomarkers to multiple bioimaging information, providing great assistance to understand the life process, benefit the disease diagnosis and manage the health of human body.

In the future, it is highly expected that DNA-mediated multi-mode analytical methods will go further into the practical clinical usage. With the steady improvement of anti-interference capability and biocompatibility, DNA-mediated multi-mode analytical methods will be applied for more complicated and various biological samples such as cerebrospinal fluid, saliva, pleural fluid, ascites, whole blood, tissue sample, etc., to obtain more abundant and accurate bioinformation. On the other hand, the non-invasive in vivo DNA-mediated multi-mode analytical methods will be another research hotline, which will benefit the achievement of the previous real-time bioinformation. Moreover, the development of combinational multi-mode methods with analytical purposes and clinical treatment functions such as PT treatment, targeted drug delivery and therapy will further enhance the significance and expand the application range from analysis to clinical treatment. Last but not least, the sensitive, fast and portable multi-mode analytical devices or instruments suitable for on-site and accurate bioanalysis will also be an essential research hot spot to greatly promote the practicability of DNA-mediated multi-mode analytical methods.

## Figures and Tables

**Table 1:** Typical applications of DNA-mediated multi-mode analytical methods for biomarkers.

Biomarkers	DNA-Mediated Strategy	Multi-Mode Analysis	LODs	Samples	Ref.
Glutathione (GSH)	Aptamer	SERS/FL	0.913 (SERS)/1.454 (FL) μmol/L	Cell	[136]
Glucose	Aptamer	PEC/EC	5.9 × 10^−5^ (PEC)/6.2 × 10^−4^ (EC) U/mL	Serum	[137]
ATP	Aptamer	Colorimetry /FL	61.29 (FL)/122.5 (Colorimetry) nmol/L	Urine	[138]
Thrombin	Aptamer	Colorimetry/EC	0.35 (EC)/10 (Colorimetry) nmol/L	Serum	[139]
PSA	G-quadruplexes	FL/SERS	0.275 (FL)/5.01 (SERS) pg/mL	Serum	[140]
Carcino-embryonic antigen (CEA)	Aptamer	EC/EC	* 0.33 (DPV)/1.67 (CV) fg/mL	Serum	[141]
Human immunoglobulin G(IgG)	Aptamer	Colorimetry/SERS	0.22 (SERS)/0.52 (Colorimetry) pg/mL	Serum	[142]
African Swine Fever Virus	DNAzyme	Colorimetry/FL	20 (FL)/20 (Colorimetry) copies/mL	Serum	[143]
Pathogen (*Vibrio parahaemolyticus*)	Aptamer	Colorimetry/EC	30 (Colorimetry)/ 5 (EC) CFU/mL	milk	[144]
microRNA	DNAzymes	ECL/EC	54.3 (ECL)/78.6 (EC) amol/L	Cell	[145]

* DPV refers to differential pulse voltammetry mode; CV refers to cyclic voltammetry mode.

## Data Availability

The data presented in this study are available on request from the corresponding author.
